# Safety Concerns of Skin, Hair and Nail Supplements in Retail Stores

**DOI:** 10.7759/cureus.9477

**Published:** 2020-07-30

**Authors:** Ariadna C Perez-Sanchez, Emily K Burns, Veronica M Perez, Evelyne K Tantry, Sahana Prabhu, Rajani Katta

**Affiliations:** 1 Internal Medicine, University of Texas Health Science Center at San Antonio, San Antonio, USA; 2 Dermatology, Baylor College of Medicine, Houston, USA; 3 Public Health, Texas A&M University, College Station, USA; 4 Dermatology, Rice University, Houston, USA; 5 Dermatology, University of Texas Health Science Center at Houston, Houston, USA; 6 Internal Medicine, Baylor College of Medicine, Houston, USA

**Keywords:** dietary supplements, supplement, human skin, female pattern hair loss, male pattern hair loss, nail diseases, us fda, biotin, drug interaction

## Abstract

Background: Dietary supplements promoted for “skin, hair, and nail” health are becoming increasingly popular, although there is a lack of regulatory oversight. As no centralized database or repository for these supplements is available, the aim of this study was to provide an overview of supplements in a sample of retail stores, with a focus on safety concerns.

Methods: Dermatology supplements were defined as those that featured the words “skin”, “hair”, “nails”, “beauty”, or “glow” in the product name or tagline. Seven stores including drug, grocery, department, and cosmetics stores were surveyed within a three-mile radius. Data were extracted from the Supplement Facts label of each product.

Results: A total of 176 separate supplements were identified, containing a total of 255 distinct ingredients. These included vitamins, minerals, food extracts, botanicals, animal products (collagen, fish oils), amino acids, a hormone, and distinct microbial strains.

Conclusion: This survey of “dermatology” supplements available in local retailers raised several safety concerns, including potential interactions, teratogenicity risks, a lack of independent third-party testing, lack of warning labels, and nutrient “overdosing”. Given limited regulation of dietary supplements, it is imperative that physicians educate patients on the potential risks. These include risks related to supplement ingredients and dosages, as well as risks related to the lack of regulatory oversight. Patients must also be educated about the multiple gaps in our knowledge of dietary supplements, especially in terms of efficacy and long-term safety.

## Introduction

Skin, hair, and nail supplements, also known as dermatology or beauty supplements, are becoming increasingly popular. Studies estimate that over 50% of the US population take some form of a dietary supplement [1]. The global beauty supplements market specifically was valued at approximately $3.5 billion in 2016, and is expected to reach almost $7 billion by 2024 [2]. This rise is attributed in part to social media marketing and the use of celebrity endorsements.

In fact, such involvement often extends far beyond endorsements: actresses, reality TV stars, social media influencers, models, and makeup companies have all developed their own lines of “beauty” supplements [3]. Minimal regulation of the dietary supplement industry by the United States Food and Drug Administration (FDA) allows a low barrier to entry for the sale of new supplements. Any individual or company can bring a new supplement to the U.S. market, with no need for proof of safety or efficacy prior to sale [4].

Manufacturers have significant leeway when choosing ingredients. The regulatory framework for dietary supplements, created in 1994, is known as the Dietary Supplement Health and Education Act (DSHEA) [5]. This act specifically “grandfathered” in any ingredients that were in use prior to passage of the law, although there is no authoritative list of pre-1994 ingredients. Also, use of an ingredient prior to 1994 does not guarantee safety. Ephedrine-containing supplements, for example, were banned in 2004 due to many reported adverse events [5].

No centralized database or repository exists to document supplements available on the market. Our goal was to document the number and variety of dietary supplements marketed as skin, hair, nail, and beauty supplements available in local retailers. We sought to determine the types and doses of ingredients utilized in these products as well as identify any potential safety concerns. 

## Materials and methods

**Figure 1:**
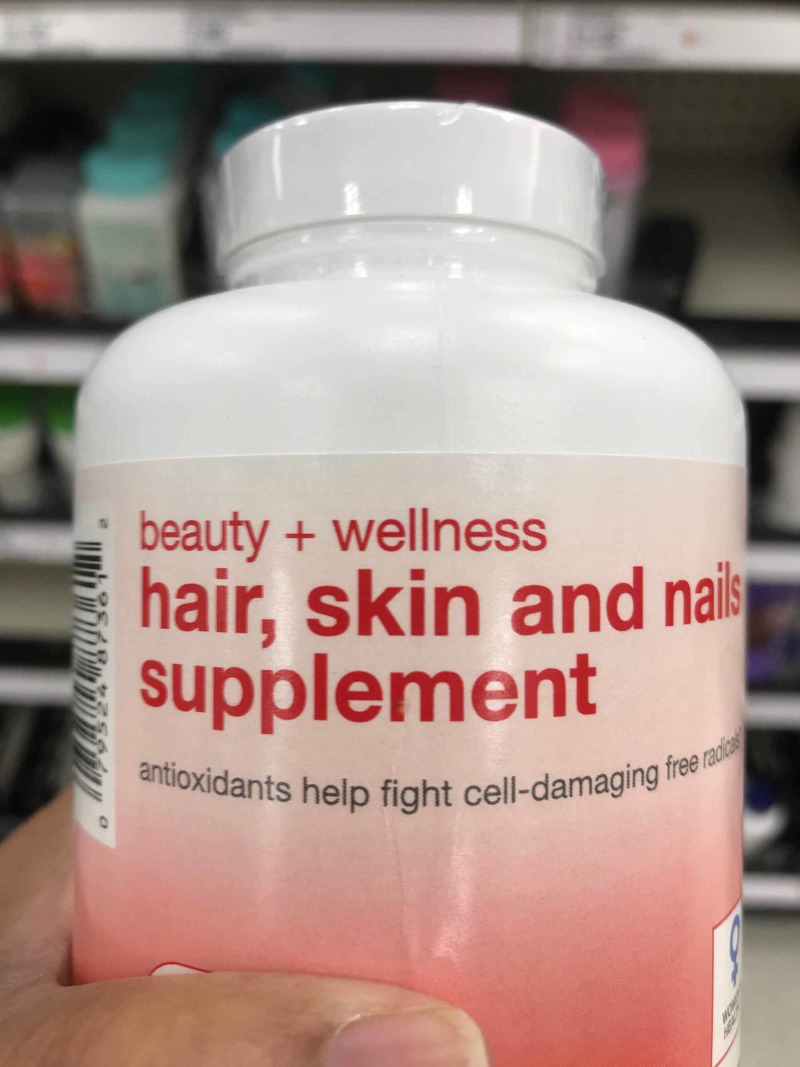
Skin, Hair, and Nail Supplement Example

Dermatology supplements (Figure 1) were defined as those that featured the words “skin”, “hair”, “nails”, “beauty”, or “glow” in the product name or tagline. Seven stores including drug, grocery, department, and cosmetics stores were surveyed within a three-mile radius using a dermatology practice in Houston as a point of reference. Data were extracted from the label of each product between August and December of 2019.

## Results

A total of 176 separate supplements were identified.

Ingredients and formulations

A total of 255 distinct ingredients were identified on the Supplement Facts labels of these products (Table 1). A total of 14 vitamins and 15 minerals were included. A total of 188 unique food extracts and botanical ingredients were identified; 38 other substances, including 10 distinct microbial strains, animal products (fish oils, collagen), amino acids, and one hormone were included. We identified 59 collagen products in total but as many products did not specify the collagen source or type, these are all included under the one ingredient “collagen”.

**Table 1:** Active Ingredients in Supplements MSM, methylsulfonylmethane; EPO: evening primrose oil; ALA: alpha linoleic acid; GLA: gamma linoleic acid

Category	List of Active Ingredients
Vitamins (In descending order of frequency of use)	Biotin/B7, ascorbic acid/vitamin C, thiamine/vitamin B1, tocopherol/vitamin E, pyridoxine/vitamin B6, pantothenic acid/vitamin B5, vitamin A, riboflavin/vitamin B2, niacin/vitamin B3, cobalamin/vitamin B12, folate/vitamin B9, vitamin D, folic acid, niacinamide
Minerals (In descending order of frequency of use)	Zinc, calcium, copper, selenium, magnesium, chromium, iron, manganese, iodine, potassium, phosphorus, chloride, sulfur, molybdenum
Other ingredients (38 total. Only selected examples listed, in descending order of frequency of use)	Collagen, silica, MSM, keratin, L-methionine, L-cysteine, melatonin, lutein, choline present in <1%: L-histidine, caffeine, EPO, fish oil, chondroitin, ALA, GLA, oleic acid, linoleic acid, L-lysine
Food extracts and botanicals (188 total. Only selected examples listed)	Selected examples: acai, amla fruit, apple cider vinegar, borage seed oil, Boswellia, flaxseed oil, gotu kola, green tea extract, ashwagandha, organic Chlorella algae, organic Moringa (leaf), pepper extract, pine bark extract, pomegranate extract, propolis, saw palmetto berry extract, Spirulina, turmeric, Bacillus coagulans, Bifidobacterium lactis, Lactobacillus rhamnosus

A wide range of formulations were utilized, including tablets, gummies, capsules, chews, chocolates, powders, softgels, and liquids.

Dosing

A number of products contained vitamins and minerals at doses far exceeding the FDA-recommended daily value (DV) (Table 2).

**Table 2:** Supplement Facts Labels Indicating Nutrients With Over 200% Recommended Daily Values

Nutrient	Labeled Dose	% of Daily Value
Biotin (vitamin B7)	10,000 mcg	33,333%
Vitamin B12	850 mcg	14,167%
Riboflavin (vitamin B2)	50 mg	3,846%
Thiamin (vitamin B1)	30 mg	2,500%
Niacinamide	500 mg	2,500%
Vitamin B6	50 mg	2,500%
Vitamin E	134 mg	893%
Chromium	200 mcg	571%
Vitamin C	500 mg	556%
Vitamin A (beta-carotene)	25,000 IU	500%
Selenium	200 mcg	363%
Zinc	30 mg	273%

Seals of quality

Only six products (3.4%) displayed seals indicating third-party testing by recognized organizations including U.S. Pharmacopeial Convention (USP) and the National Sanitation Foundation (NSF).

Warning labels

In our sample, a total of nine biotin (vitamin B7) supplements contained doses over 10,000 mcg. None listed a warning on potential interactions with laboratory testing.

No supplement contained information on tolerable upper intake levels (UL), the need to consider dietary sources of nutrients, or pregnancy warning categories. 

## Discussion

In our survey of products advertised as dermatology supplements, we identified a total of 176 separate supplements, utilizing 255 distinct ingredients encompassing a wide array of substances. Legally, there is no definition of what constitutes a “skin, hair, and nail” supplement. The legal definition of a dietary supplement, per the FDA, is that of an ingestible product containing a “dietary ingredient” but one that is not marketed as a conventional food or “sole item of a meal or diet”. A “dietary ingredient” can consist of a vitamin, mineral, herb or botanical, amino acid, dietary substance intended to increase the total dietary intake, or a “concentrate, metabolite, constituent, extract, or combination of the aforementioned items” [5]. This definition is broad enough to include a wide range of ingredients, with over 85,000 supplements reported utilizing multiple combinations of ingredients [6]. 

Supplements are regulated by the FDA as foods, not drugs, which has multiple safety implications. There is no need for manufacturers to prove safety or efficacy prior to sale [4]. There is no restriction on doses of vitamins or minerals that may be used, even for nutrients with defined tolerable ULs. Manufacturers may mix and match ingredients and combine them into a supplement, without the need to document a lack of interactions between ingredients and/or other medications. While companies are expected by the FDA to adhere to good manufacturing practices, no proof is required [4].

Our survey of skin, hair, and nail supplements highlights several safety concerns, including potential interactions, teratogenic effects, drowsiness, nutrient “overdosing”, allergenicity, quality concerns, and risks related to unknown factors.

Drug interactions

The potential for supplements to interact with prescription medications, other supplement ingredients, and even laboratory tests is well documented. One literature review documented more than 1,400 unique interactions involving over 200 supplements and herbs [7]. Of concern is that many potential interactions have yet to be investigated or described.

As one example, biotin (vitamin B7) has a long history of use in “nail” and “hair” supplements, yet it was only in 2017 that the FDA issued a warning about interactions between biotin and certain laboratory tests, including those that test for cardiac function and thyroid function [8]. This warning was based on a study of human volunteers consuming 10,000 mcg of biotin daily for one week. Notably, of the nine biotin supplements in our sample that contained doses more than 10,000 mcg, none warned of this interaction.

Teratogenicity

Current labeling laws do not require pregnancy category warnings on any supplements, even for those ingredients that present known teratogenicity risks such as high-dose vitamin A or saw palmetto. Every supplement in our sample cautioned pregnant women to seek medical advice prior to use. (“If you are pregnant, nursing, taking any medications, or have a medical condition, consult your doctor before use.”) However, no supplement included pregnancy category warnings, which are required on prescription teratogenic medications.

Saw palmetto was found in a “hair” supplement. This botanical inhibits 5-alpha-reductase [9,10]. 5-Alpha-reductase inhibitors administered to pregnant animals are associated with abnormal genitalia in male offspring. These drugs are therefore required by the FDA to be labeled as pregnancy category X [11]. No such requirement is in place for supplements.

Also of concern are unknown teratogenicity risks. For many nutrients, risks related to high-dose consumption have not been studied systematically. Many supplements in our sample contained zinc, which warrants further study, as one study noted an association between elevated levels of zinc in umbilical cord blood and adverse neonatal neurobehavioral development [12].

High-dose vitamin A is another concern. In a study of pregnant women who averaged more than 10,000 IU per day of vitamin A orally (in the form of retinoid compounds), approximately one in 57 had a fetal malformation owing to the supplement [13]. This risk is considered particularly high before the seventh week of pregnancy, when some women may not know that they are pregnant. In our sample, one product contained 25,000 IU of beta-carotene. Beta-carotene is a precursor to vitamin A. While the teratogenic risk of supplemental beta-carotene is not known, it is considered equivalent to preformed retinoids when calculating retinol activity equivalents (RAE) [14]. Therefore, further studies of the teratogenic risk of high-dose beta-carotene are warranted.

Drowsiness

Seven supplements in our sample contained melatonin, a hormone that may cause drowsiness. One product did not warn of this side effect.

Nutrient “overdosing”

The risk of nutrient “overdosing” is a significant concern for many vitamins and minerals. In our survey, manufacturers utilized highly variable dosing for many nutrients. Doses of biotin, for example, ranged from 100% DV to 33,333% DV. In fact, multiple products contained vitamins and minerals at doses far exceeding the DV (Table 2).

Consumers may not realize that some vitamins and minerals are harmful if chronically consumed at doses higher than that provided by food sources. Chronic high-dose supplementation of certain vitamins and minerals has been linked to neuropathy, gastrointestinal disturbances, vasodilation, and milk-alkali syndrome [15]. These risks led to the development of tolerable ULs. ULs have been established for 24 vitamins and minerals, and are considered to be the highest "usual intake level" of a nutrient that poses no risk of adverse effects. 

ULs encompass nutrient intake from all sources, including foods and supplements. These limits were established because a significant proportion of the population regularly consumes either “large amounts of highly fortified foods, large number of moderately fortified foods, or non-food sources such as nutritional supplements, or any combination of the three” [15].

No products in our sample provided information on ULs or the need to consider food sources of nutrients. Of particular concern are supplements containing vitamins and minerals that either exceeded the UL or contained such high doses that additional food intake would place the user close to the UL.

One product contained beta-carotene, a vitamin A precursor, at a dose of 7,500 mcg of RAE. While the Food and Nutrition Board has not established ULs for beta-carotene, the UL for pre-formed vitamin A is 3,000 mcg of RAE per day, and the FNB does not recommend beta-carotene supplements for the general population except as a source to prevent vitamin A deficiency [14]. Beta-carotene supplementation has been linked to an increased cancer risk in male smokers: in one randomized controlled trial (RCT), male smokers taking a beta-carotene supplement had an increased risk for lung cancer as compared to placebo [16].

Another product contained selenium at a dose of 200 µg. If also consuming selenium-rich foods, consumers could surpass the UL of 400 mcg. A single Brazil nut, for example, contains 90 mcg [17]. Excess selenium intake (with supplement use) has been linked to higher risk of diabetes and even higher overall mortality rates [18,19].

Risks of adverse, long-term health outcomes exist, even for supplement use not exceeding the UL. High-dose vitamin B6 and B12 supplements have been linked to higher risk of lung cancer in smokers. In one study, male smokers consuming greater than 20 mg of B6 via supplements over 10 years were three times more likely to develop lung cancer. One “hair loss” supplement in our sample contained 50 mg of B6. In the same study, male smokers consuming more than 55 mg of B12 were four times more likely to develop lung cancer. One “hair loss” supplement contained 850 mcg of B12 [20].

The use of other high-dose nutrients has not been studied systematically, so further unknown risks may exist. One study documented this problem. In this RCT, researchers evaluated a supplement that appeared promising for the reduction of skin cancer. This supplement contained vitamin C, vitamin E, beta-carotene, selenium, and zinc. Unfortunately, after median follow-up of 7.5 years the incidence of skin cancer was actually higher in women ages 35-60 years consuming this supplement as compared to placebo [21]. Given the lack of FDA regulation, RCTs such as this one are warranted and may reveal further concerns.

Allergenicity

Among collagen products, 17% indicated sourcing from “fish”. Of these, 90% lacked allergen warnings. Although the allergenicity of collagen products derived from seafood has not been studied systematically, hydrolyzed fish collagen has been reported to cause anaphylaxis [22].

Quality concerns

One potential factor accounting for the significant growth of the dietary supplement market is considered to be the low barrier to entry. Companies are “expected to adhere to”, but do not have to prove, good manufacturing practices [4]. 

An FDA spokesperson was quoted in an article highlighting the agency's limited resources. "…with a staff of less than 24 people to regulate an industry worth an estimated $36.7 billion, the agency has focused its resources on cracking down on unsafe products, said Meyer" [23].

Multiple quality issues with supplements have been reported. Consumer Lab, an independent investigative laboratory, evaluated a total of 15 collagen supplements and found that one product contained unacceptably high levels of cadmium, a heavy metal [24]. Other quality concerns include doses that differ markedly from those indicated on the label, dosing inconsistency across products, and issues with disintegration of tablets [25]. Other issues include contamination with microbes or adulteration with heavy metals or prescription medications.

While factories are sometimes inspected by the FDA, the agency is only able to inspect a small fraction. Consumer Lab reported that in fiscal year 2019, the FDA investigated a total of only 560 factories, and issued letters of non-compliance to over 50% of these [26]. The list of manufacturers receiving citations included some that produce collagen powders and hair loss supplements.

Consumers of supplements may be advised to seek products that have undergone third-party independent quality testing. Per the Office of Dietary Supplements (ODS) of the NIH, “seals of approval” may be displayed on a supplement, indicating testing by an independent organization [27]. These seals indicate that the product contains the ingredients listed on the label and does not contain harmful levels of contaminants. Three such organizations listed by the ODS are USP, NSF, and Consumer Lab.

Only six supplements (3.4%) in our sample displayed such seals. Five displayed the USP seal, all from the same manufacturer, and one displayed the NSF seal. While two other products displayed seals, one simply listed “third-party purity testing”, and the other was tested by a manufacturer-funded research lab.

The use of these seals was not related to price of the supplement.

The most common formulations in our survey were tablets, capsules, and gummies. Gummies are particularly concerning: Consumer Lab, a third-party investigative laboratory, has reported that gummies are the most likely formulation to experience dosing inconsistencies [28]. None of the gummies in our study contained seals from third-party laboratories. 

Unknowns

Since no studies need be conducted on any of these products prior to use, we have limited information on short-term and long-term risks. 

Apart from the unknown risks of substances such as herbals, animal products, and microbes, even vitamins and minerals pose unknown risks. As discussed, some of these risks have been discovered “accidentally”, as with cancer prevention RCTs of vitamins and minerals (at supraphysiologic doses) that were actually linked to a higher risk of cancer [16,21].

The unknown aspects of the collagen manufacturing process warrant further consideration. Over half of supplements containing collagen lacked information on source. Consumers should be advised to seek answers from manufacturers on several important questions: What animals and animal parts (or combination thereof) served as the source? What measures were used to protect against contamination or adulteration? If sourced from fish, were low-mercury fish used? If sourced from cows, what steps were taken to ensure that no brain or nervous system matter was included, in order to prevent prion disease? 

Limitations

As our study was designed to investigate products sold in local retailers, this represents only a limited sample of such products. Further research should evaluate online products and a wider geographic region.

Future directions

We encourage strengthened regulation and oversight of the supplement industry, particularly in terms of ensuring quality and safety of ingredients and doses used. Pregnancy category warnings, especially for category X substances, should be required. As many consumers may not recognize the risks of “mega-dose” vitamins and minerals, we encourage labeling stating that such high doses should only be taken by those with deficiency. Further research on efficacy, side effects, and teratogenicity risk is required.

Consumers at a minimum should seek products that contain third-party “seals of approval” that ensure a product contains the ingredients and doses listed on the label and is free of contamination and adulteration.

Finally, with no required post-marketing surveillance programs, it is imperative that physicians and consumers report any possible supplement reactions. This may be done by contacting the consumer complaint coordinator or filing a safety report online through the Safety Reporting Portal [29].

## Conclusions

Given limited regulation of dietary supplements, it is imperative that physicians educate patients on the potential risks. These include risks related to supplement ingredients and dosages, as well as risks related to the lack of regulatory oversight. Patients must also be educated about the multiple gaps in our knowledge of dietary supplements, especially in terms of efficacy and long-term safety.
